# Professional Conscience

**Published:** 1905-05

**Authors:** Richard Douglas

**Affiliations:** Nashville, Tenn.


### PROFESSIONAL CONSCIENCE.*

By RICHARD DOUGLAS, M.D., Nashville, Tenn.

As a representative of the general profession of medicine, I have been assigned the pleasing duty of welcoming you into the full fellowship. The diplomas you have just received attests to your proficiency in all branches of medicine and surgery.

After four years of assiduous study, you have been tested and found worthy of the honor of the degree *Doctor Medicinae.* I cordially greet you upon this momentous occasion. This vast audience, composed of the elite of this wealthy and aristocratic "Queen City of the South" has assembled in your honor. Do not permit the ceremonies of the occasion to dazzle you; be not too complacent nor self-congratulatory. This hour marks a crisis in your lives.

Upon occasions of this nature, it is customary for the speaker to encourage you in borrowed eloquence, with pictures of a glorious future; assuring you of success; promising you all the rewards and emoluments of a large practice, if you will only be diligent and persevering. This is one of the professional lies of which I will have more to say later.

The road to success in medicine is a long and dreary one, beset with many bogs, in which many of you may mire and sink into oblivion. Some will travel it to moderate success; perhaps one or two may even hope for fame. At least six per cent. of the class, according to statistics, will abandon the effort and will seek their living in other channels. It is well known, that when a man fails in his effort to be a doctor, he not infrequently takes to swapping horses.

To those of you who are in earnest and have undertaken the study of medicine for the welfare of humanity, without the hope of personal gain, to you I trust to have something to say which may prove helpful.

Many years ago I had the honor of being a private student of

* Read before the graduating class of the College of Physicians and Surgeons, Atlanta, Ga.

that great surgeon and sage, Samuel D. Gross. Once, while in familiar conversation with him, wishing to know how to conduct myself when I entered practice, I inquired, "Professor, when I call in a consultant, what shall I tell him of the case?" His reply was brief: "Tell him all and the truth."

Recently there appeared in one of our medical journals, an article by Cabot, of Boston, condemning the "Professional Lie." Lately, in conversation with William Mayo, of Rochester, Minn., recognized the world over as one of our greatest living surgeons, speaking of surgeons generally, he said: "The great surgeon of the future will not be the most skillful operator, nor yet the most astute diagnostician, but the man who conducts his work honestly and solely for the welfare of his patient."

These men had attained the highest professional eminence and were impressed with the need and wisdom of honesty. It seems appropriate, therefore, that I should take this occasion to impress upon you, young gentlemen, this essential to success in life; and what I shall say to you is upon the subject of *professional conscience.*

"Conscience is supposed to be a compound of faculties by which we decide upon what is called rightness or wrongness of acts." Herbert Spencer says, "Conscience is called fallable, educable and vacillating." John Foster, in his celebrated essay, thus speaks of it. "Among human possessions, there is nothing so absurd and chimerical as conscience; it is a bundle of habits." Von Humboldt said that every man, however good, has a yet better man within him. When the outer man is unfaithful to his deeper convictions, the hidden man whispers a protest. The name of this whisper in the soul is conscience. (Hillis.)

I assume that conscience is a normal attribute, susceptible of growth and refinement under cultivation. That education sharpens our perceptions and renders us more capable of accurately differentiating right from wrong. Though conscience is difficult to define, it is, nevertheless, quite clear to us all what is meant by the difference between the truth and a lie.

Bacon says, "It will be acknowledged even by those who practice it not, that clear and round dealing is the honor of man's nature; and that a mixture of falsehood is like alloy in coin of gold and silver, which may make the metal work better, but embaseth it." "While to say that a man lies," says Montaigue, "is as much as to say that he is brave toward God and a coward toward man. While with a lie he faces God, he shrinks from man."

Conscience is inseparable from duty in medicine; one impels the other. If, by knowledge you know your duty, your conscience directs you to discharge it. A duty conscientiously done is always well done. Indeed, I may go further and say that our conscience is the whetstone of our energies. When confused or uncertain, conscience seeks knowledge for guidance. Living under the dictates of an enlightened conscience is the most exalted state of man.

Professional conscience, as applied to our calling—medicine—means simply, honesty in your professional conduct. Be honest with your patient, with your professional associates, true to your science and yourself, and "It must follow as the night the day, thou canst not then be false to any man."

Selfishness, indolence, ignorance and inaccuracy are the evils that tempt our professional conscience. Selfishness is manifested chiefly by a jealous fear that some brother doctor may win favor in the eyes of your patients or his friends, and a much-needed consultation is refused or delayed until too late. There are men who shrink from having their work inspected or refuse to have assistance from an honorable consultant, fearing that something may be done for the patient's good which will redound to the credit of the consultant.

To a lay audience, I am ashamed to confess that this spirit may be found in the profession. It is a pity that it is true; but thank God, this craven, this wolf, is not long in disguise. A few exhibitions of such narrowness reveals this man with a dead conscience. The public is not misled; his career, always inglorious, is a short one.

In the nature of a true doctor, selfishness has no part. A noble, self-sacrificing generosity characterizes every act of his professional life. For love of science and humanity, he surrenders the dearest relations in life; he is a stranger in his own home and finds small occasion for social pleasures. His purse, never too wellfilled, is constantly open to the demands of charity; his daily life is full of good deeds.

Since conscience evolves out of knowledge, ignorance practically excludes conscience. In the judgment we pass upon one another, we measure responsibility not by the thing done, but by the opportunity one has of knowing better or worse. So of you, the people will say, "This man has had opportunities, advantages. The medical faculty of the renowned College of Physicians and Surgeons have declared him to be a capable physician, and we will try him."

Now, it remains for you to bring honor or dishonor upon your *Alma Mater.* Your opportunities have been the best; your responsibilities will increase with each to-morrow; your attainments suffice for the present occasion, but are wholly inadequate for the perplexities of the future. Minerva is a fleet and impatient goddess. If you are to travel with her she will not wait for you. If she leaves you, overtake her you can not. Your one hope is in keeping pace with her.

In your work constantly interrogate yourself. Be your own quiz master. If you are not master of the case in hand and have the means of becoming so and do not resort to them, then you are culpably ignorant; you are not acting conscientiously. Some one will say to you, "Thou fool," and you will be in danger of professional extermination which is to a doctor, eternal damnation.

Ignorance in a doctor concerning those things which he *should* know, and pretends *to* know, brands him at once a counterfeit, a cheat and impostor. He appears in so many disguises that he may escape recognition. A very familiar type is Dr. Pomposity. Not infrequently he resembles very closely a minister; his garb is austere, with dignity so precise and manner mild. He is wisely silent in all places save church or Sunday-school. There he is conspicuously devotional. This crafty cheat may seek professional advancement by political and business alliance; occasionally he will presume to report some most unusual, unique case.

I have known him to actually publish articles in reputable journals, claiming to have performed certain surgical feats without having met with the conditions described. It is not worth while to study this polymorpheus individual. Whatever shape he may assume, he is a conscienceless quack; he is not of our flock, and should be branded and placed with the goats.

Indolence lulls professional conscience to sleep. It threatens the career of every young physician. The tendency to loaf, the long undisturbed office hours, the pleasures of social life, all lure him from his work. Idealized indolence is exemplified in the young doctor who marries a rich woman. He becomes a mere pensioner and is content that she furnish "bed and board."

The young man just starting in practice indolently allows opportunities to escape him. He receives a call. "It is only a sick pauper, it is of no consequence. Why respond to it; there is no pay in it anyway," he will argue, and what a mistake. There is much in *every* case. It is an unread book; open and find therein a golden lesson. And as for the fee, it too, may be had, and from every pauper—experience, a rich fee. Hasten to collect it, for your future drafts will be heavy upon it. How helpless you will be without this reserve fund. In times of panic when others are ill at ease and uncertain, you can draw upon this fund of experience. It is knowledge first-hand and can be relied upon.

One may be professionally indolent, without necessarily being idle. Indeed, he may be very busy playing the flute, courting, or employed in some equally diverting and unprofitable pastime.

Osler, an authority on everything in medicine, save the age limitation of man's usefulness, says, "Work is the master word in medicine." Forget for one hour the task that is before you, relax for ever so short a time and you are as worthless to the body of science as the rear stragglers are to a besieging army. Most appropriate is the thought so beautifully expressed by Horace Mann: "Lost, yesterday, somewhere between sunrise and sunset, two golden hours, each set with sixty diamond minutes. No reward is offered, for they are gone forever."

Inaccuracy, a very serious fault, may at first be mere carelessness. But the habit grows so that it may lead to absolute irresponsibility. Doctors fall into this habit, either through inattention to detail, or else by yielding to an inclination to boast. We hear them talking after this manner: That they are so busy, so tired, so worn out that they must excuse themselves from their patients. They have just completed their one hundred cases of appendicectomy without a death; they make forty calls a day, and a few other such exaggerated statements. These same men speak, act and live in the superlative degree, and in their talking of their cases they picture them in the most highly colored and exaggerated way. Such statements must be attributed either to errors in observation or to the desire to mislead. I frankly believe that these are unintentional, professional lies; but they render the perpetrator a very uncertain factor in medicine.

In illustration, if this form of lie admit of personal allusion. Many years ago I had performed a certain operation nineteen times. In speaking of it to brother physicians, I was in the habit of saying nineteen or twenty times. I soon forgot the nineteen and found it easier to say twenty, and repeated the statement so often that I really believed that I had performed the operation twenty times. It was only after referring to my records that I undeceived myself and discontinued the lie. It was in connection with this same operation that I yielded to the temptation to boast, in the presence of a very capable English surgeon, Mr. Doran. "Oh," said I, "I have done that operation twenty times." "Ah," said he, "you Americans do a lot of surgery, some of it very bad, don't you know." I have tried to avoid lying and boasting since those days, twenty years ago.

Inaccuracy pervades every branch of our profession. Laboratory men are, perhaps, by training, more thorough and exact than the rest of us. Of what value is judgment based upon insufficient and inaccurate evidence? All men who make hurried, superficial examinations are inaccurate and necessarily a little careless with the truth. The painstaking, thorough men, perhaps of less capacity, will discover the error of oversight and subject the less careful brother to ridicule. Therefore, let me warn you, in all your professional work, either as principal or consultant, gather your own histories, make your own physical examination of the patient, not that you should mistrust others, but it is a maxim as staunch as Standish himself, that "If you wish a thing well done, do it yourself."

Inaccuracy becomes a lie, when you wilfully mislead yourself or others. Dangerous to the profession is that man who inaccurately publishes his work, and, if I mistake not, the spirit of distrust is growing in our ranks. Within our day the work of the ablest and best men has been questioned. How utterly debased and wicked is that man who would mislead his coworkers by a malicious misstatement. We can not estimate the evil consequences. Therefore, keep your conscience sensitive and alive to the obligatious to your science, not alone by the earnestness and character of your work, but by the accuracy with which you record it.

Our medical knowledge, indeed the science of medicine, consists largely of facts and phenomena, known to occur in sufficient frequency when certain antecedents are uniformly followed by certain consequences, thus furnishing a basis by which we may logically conclude that similar phenomena attend certain definite conditions. How invaluable it is that our observations and records should be accurately taken, since it is the combined work of us all that writes history for the guidance of the future generations.

Your professional conscience is taxed according to the multiplicity of problems that are presented. When the practice of medicine was mere empiricism, duty, as understood, was very simple and clear; but now, that it has become a science, fast assuming exactness in all lines, so much is required of us, so much is known, that we can not feel that we have conscientiously discharged our duty unless we apply with exactness and care every known method in diagnosis and treatment. In former years, a few symptoms were sufficient for diagnosis. To observe these met the physician's obligations in the case. How different now! Our science has precise methods, evolved by clinical work and experimentation.

You have been taught these methods; it is your duty to apply them in your practice. If you fail to do so, I ask, if you have rendered your patient conscientious service; that is, given the benefit of all that modern medicine can offer? Your neglect to do so renders you culpably ignorant or indolent.

"New occasions teach new duties,
Time makes ancient good uncouth;
They must upwards still, and onwards,
Who would keep abreast with Truth."

A genius has but small place in medicine. Such intellects are not generally useful. The safe, good doctor is the one with patience and energy, who tirelessly and painstakingly enters deeply into every question after our well-recognized methods. "Shakespeare tells us that all the clouds give in rain they got in mist, which is the poet's way of saying that what he gave in inspiration he got by way of perspiration." (Hillis.)

Your life is one of preparation. Disease is presented to you in all its varied phenomena. Painstaking, analytical study is constantly required, that you may so group its symptomatology and obtain a logical diagnosis. There is scarcely one of you who has not been dazzled by the achievements in the surgical clinic the past winter. The marvelous cures obtained by the wizard hand of the surgeon seemed well-nigh miraculous to you. The direct methods and positive results appealed to your active minds; and I am quite safe in declaring that half of you expect to become surgeons. You see the *object attained* and not the *means*.

Are you aware that the skilled surgical operations that you have witnessed this winter required years of study and preparation to acquire that knowledge necessary to guide the surgeon's hand? The courage that emboldened him to attempt a grave operation was born of conscientious preparation.

The first test of your conscience is the earnestness and trueness with which you have prepared yourself for your life's work, and I am authorized by your faculty to publicly declare that this you have done excellently well. When Nestor stood forth before the Greek generals and counseled an attack upon Troy, he said, "The secret of victory is in getting a good ready." You are about to lay siege to the *public confidence*, more securely entrenched than Troy within her walls, and containing a treasure far richer than the flippant Helen. You have made a good ready, and the fight is on. All I ask is that you fight boldly and truly, which means to study diligently, to observe accurately and record honestly. Let the public see that these are your methods, and they can not withhold from you their confidence and patronage. The very singleness of your purpose will appeal to them and by and by they will recognize you as a man of force, a man of character, which means nothing more than energy and intelligence, directed by conscience. To win and retain this position, you must in all your dealings with all your patients be open, frank and honest.

Cabot tells us that there is no need of a professional lie. It is true that those who are ignorant of human nature and are timid may claim that the whole truth is cruel. This is mere sentiment. It has been appropriately said: "In matters of taste, drift with the tide; but in matters of principle, be steadfast as a rock." Are you not violating the principles of our science when you attempt to mislead a man with advanced tuberculosis by attributing his weakness and loss of flesh to trivial and curable maladies? Will not he and his friends ultimately learn the truth and know us to be either ass or knave?

Glorious as are the achievements of modern surgery, we are not yet warranted in assuring a patient that any surgical procedure is unattended by danger. Now and then it will happen to the best of us to meet with unexpected mortalities. How unenviable, then, is our position, if we have assured against danger. There are times when the patient's interest and your own seem somewhat opposed. Your conscience should ever guide you aright; there can be no quibbling in matters of this kind; your duty, however self-sacrificing it may appear, is solely for the welfare of your patient. If conscientiously performed, it may mean temporary embarrassment and discredit to you, but ultimately right will prevail.

If my words have been serious to-night, believe me they have not belied my feelings. Constant association for twenty-five years with young men in medicine, has made them dear to my heart. I sympathize with you in all your fears and difficulties and enter heartily into your hopes and ambitions and glory for you in the calling that you have chosen. Most men work for a living; those of a finer mold work for distinction in business, church or state affairs, while you, in humble imitation of Him who walked upon the shores of Galilee, work for the love and welfare of your fellow man. There is no higher or nobler calling.

